# Factors Associated With Intubation and Prolonged Intubation in Hospitalized Patients With COVID-19

**DOI:** 10.1177/0194599820929640

**Published:** 2020-05-19

**Authors:** Kevin Hur, Caroline P. E. Price, Elizabeth L. Gray, Reeti K. Gulati, Matthew Maksimoski, Samuel D. Racette, Alexander L. Schneider, Ashoke R. Khanwalkar

**Affiliations:** 1 Department of Otolaryngology–Head and Neck Surgery, Feinberg School of Medicine, Northwestern University, Chicago, Illinois, USA; 2 Biostatistics Collaboration Center, Feinberg School of Medicine, Northwestern University, Chicago, Illinois, USA

**Keywords:** COVID-19, SARS-CoV-2, coronavirus, hospitalized, adults, mechanical ventilator, intubation, tracheostomy

## Abstract

**Objective:**

To identify risk factors associated with intubation and time to extubation in hospitalized patients with coronavirus disease 2019 (COVID-19).

**Study Design:**

Retrospective observational study.

**Setting:**

Ten hospitals in the Chicago metropolitan area.

**Subjects and Methods:**

Patients with laboratory-confirmed COVID-19 admitted between March 1 and April 8, 2020, were included. We evaluated sociodemographic and clinical characteristics associated with intubation and prolonged intubation for acute respiratory failure secondary to COVID-19 infection.

**Results:**

Of the 486 hospitalized patients included in the study, the median age was 59 years (interquartile range, 47-69); 271 (55.8%) were male; and the median body mass index was 30.6 (interquartile range, 26.5-35.6). During the hospitalization, 138 (28.4%) patients were intubated; 78 (56.5%) were eventually extubated; 21 (15.2%) died; and 39 (28.3%) remained intubated at a mean ± SD follow-up of 19.6 ± 6.7 days. Intubated patients had a significantly higher median age (65 vs 57 years, *P* < .001) and rate of diabetes (56 [40.6%] vs 104 [29.9%], *P* = .031) as compared with nonintubated patients. Multivariable logistic regression analysis identified age, sex, respiratory rate, oxygen saturation, history of diabetes, and shortness of breath as factors predictive of intubation. Age and body mass index were the only factors independently associated with time to extubation.

**Conclusion:**

In addition to clinical signs of respiratory distress, patients with COVID-19 who are older, male, or diabetic are at higher risk of requiring intubation. Among intubated patients, older and more obese patients are at higher risk for prolonged intubation. Otolaryngologists consulted for airway management should consider these factors in their decision making.

The SARS-CoV-2 virus, more commonly known as coronavirus 2019 (COVID-19), is a novel respiratory virus that was first recognized in China and has now spread across the world. Existing literature has described the clinical presentation of COVID-19 to be similar yet distinct from prior comparable coronaviruses, such as Middle East respiratory syndrome and severe acute respiratory syndrome.^1^ COVID-19 appears to have a lower case fatality rate but a higher rate of transmission, leading to far more total deaths.^1,2^ Severe disease is notable for hypoxic respiratory failure requiring prolonged supportive care oftentimes involving intubation and invasive mechanical ventilation.^3,4^

In the United States, the rapid transmission of the virus, which can also be spread by asymptomatic individuals, has led to a sharp increase in infections in a short period, straining the health care system. Of significant national concern is the limited supply of mechanical ventilators and the number needed to adequately satisfy the demand from the US population.^5^ However, risk factors associated with the need for mechanical ventilation among those infected with COVID-19 are unclear. While preliminary reports from the Centers for Disease Control and Prevention suggest that >70% of hospitalized patients have ≥1 underlying health conditions, only 5.8% of reported cases contain information on comorbidities.^6^ Moreover, the relative importance of different underlying health conditions and sociodemographic factors in the disease course have yet to be determined from currently available studies due to inadequate adjustment for possible confounding factors.^7^

As COVID-19 infections continue to rapidly consume the US health care system’s limited resources, identifying populations at risk of a more severe disease course is critical for otolaryngologists, who may be called on to manage the airway of patients with COVID-19 in the hospital or decide which intubated patients are candidates for a tracheostomy. In this study, we aimed to identify individual risk factors associated with intubation among hospitalized patients with laboratory-confirmed COVID-19 in the Chicago metropolitan area, as well as time to extubation among intubated patients.

## Methods

### Data Source

After approval by the institutional review board at the Northwestern University Feinberg School of Medicine, data on hospitalized patients with laboratory-confirmed COVID-19 infection during or prior to admission were identified and compiled from the Northwestern University Enterprise Data Warehouse, an integrated repository of clinical data for Northwestern-affiliated health care centers.^8^ A confirmed case was defined as a positive result on a reverse transcriptase polymerase chain reaction assay of a specimen collected by a nasopharyngeal swab. Patients were included if they were aged ≥18 years and were admitted to any of the 10 hospitals in the Northwestern Memorial HealthCare system spread across the Chicago metropolitan area (see Supplemental Table S1, available online) between March 1 and April 8, 2020. Hospitalized patients with documented “do not resuscitate and do not intubate” (DNR/DNI) orders and those who left the hospital against medical advice were excluded from the study cohort. Patients who had missing data on investigated predictor variables and did not reach a clinical endpoint of intubation or discharge from the hospital were also excluded. Clinical outcomes, such as mortality, discharge, intubation, and extubation, were last recorded on April 18, 2020.

### Measures

Sociodemographic information was collected, including age, sex, race, ethnicity, and history of tobacco use. Symptom and clinical history obtained on the day of admission, including recent travel and contact with individuals infected with COVID-19, was manually extracted from the medical record. Vital signs included systolic and diastolic blood pressure, heart rate, respiratory rate, maximum temperature, lowest documented oxygen saturation, and body mass index (BMI) measured in the emergency room. Medical history included all comorbidities documented on admission.

Comorbid disease was classified into diabetes, hypertension, cardiovascular disease, organ transplantation, immunosuppression, cancer, obstructive sleep apnea, pulmonary disease, and chronic kidney disease. Cardiovascular disease included myocardial infarction, cerebrovascular accident, congestive heart failure, valvular heart disease, and arrhythmias. Pulmonary disease included asthma, chronic obstructive pulmonary disease, and interstitial lung disease. Patients with no history of the aforementioned comorbidities were classified as “none of the above.”

Chest radiographic imaging results were reviewed on the day of admission and classified as having positive findings if the radiologist documented any evidence of ground-glass opacity, consolidation, or infiltrates.

The primary outcome of this study was intubation with an oral endotracheal tube and attachment to a mechanical ventilator during the hospitalization. The secondary outcome was time to extubation, defined as the number of days from intubation to extubation.

### Statistical Analysis

Univariate analysis was performed with Wilcoxon rank sum tests for continuous variables and chi-square or the Fisher exact test for categorical variables. A random forest model was used to predict intubation by entering all collected variables into the model via repeated cross-validation with internal down-sampling to account for imbalance between cohorts. Logistic regression of selected variables of importance from the random forest model was performed to report odds ratios (ORs). Leave-one-out cross-validation was performed to assess the discrimination of the final logistic regression model. For analysis of time to extubation, the Kaplan-Meier method and log-rank tests were used to explore the relationship between baseline variables and outcome. A multivariable analysis was performed with Cox proportional hazards regression models stratified by location of hospital, in which (1) demographics and medical history were considered covariates and (2) deaths and patients still intubated at the time of last follow-up were censored. All statistical analyses were conducted with version 3.6.0 of the R programming language (R Project for Statistical Computing; R Foundation). All reported *P* values were considered significant at <.05. See the online appendix for details regarding the statistical methods.

## Results

### Demographics

**Figure 1.**
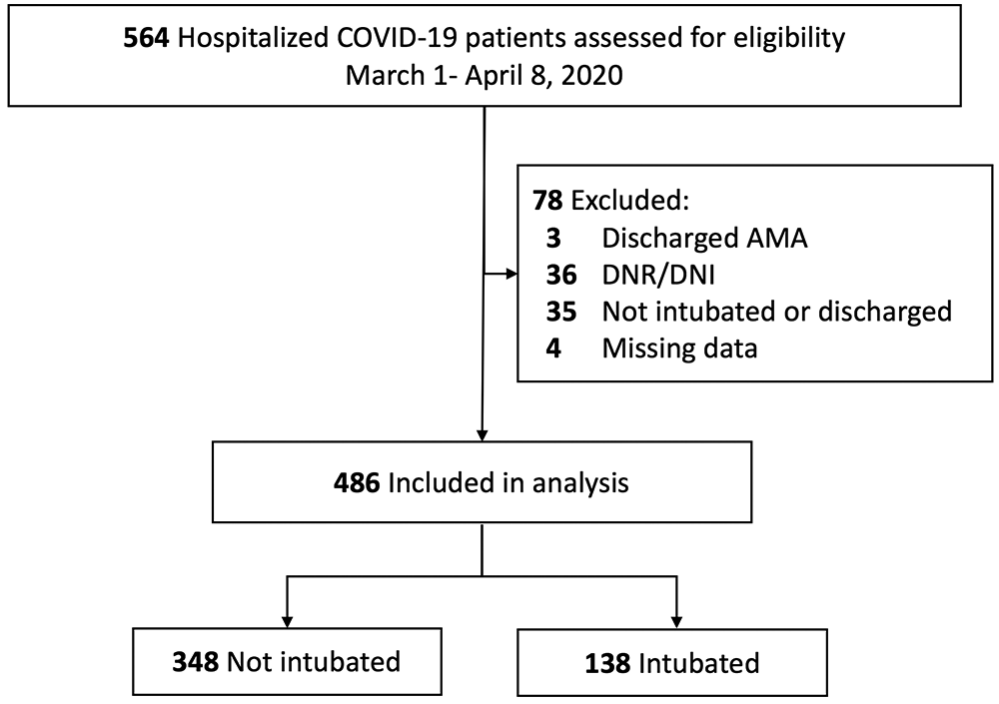
Patient selection. AMA, against medical advice; DNI, do not intubate; DNR, do not resuscitate.

This study included 564 unique hospitalized patients admitted between March 1 and April 8, 2020, who tested positive for COVID-19 on a polymerase chain reaction assay. After review of the medical records, 78 of these patients were excluded because they had a DNR/DNI order, left the hospital against medical advice, had missing BMI or smoking data, or had not been intubated or discharged at the time of censoring (**Figure 1**). A final cohort of 486 patients was analyzed with a mean ± SD follow-up of 19.6 ± 6.7 days from date of admission. The median patient age was 59 years (range, 19-101); 271 patients (55.8%) were male; 259 (53.3%) had a BMI ≥30; and 163 (33.5%) were former or current smokers (**Table 1**). The most common comorbidities were hypertension (54.9%), diabetes (32.9%), and cardiovascular disease (22.8%); 113 (23.3%) patients had no comorbidities in any of the 9 categories analyzed.

**Table 1.** Characteristics of Intubated Patients With COVID-19 Infection.^a^

		Intubated		
	Total (N = 486)	No (n = 348)	Yes (n = 138)	*P* value
Age, y				
≤60	269 (55.3)	214 (61.5)	55 (39.9)	
>60	217 (44.7)	134 (38.5)	83 (60.1)	<.001^b^
Sex				
Female	215 (44.2)	165 (47.4)	50 (36.2)	
Male	271 (55.8)	183 (52.6)	88 (63.8)	.033^b^
Race and ethnicity^c^				
Non-Hispanic White	191 (39.3)	126 (36.2)	65 (47.1)	
African American	144 (29.6)	111 (31.9)	33 (23.9)	
Hispanic white	112 (23.0)	81 (23.3)	31 (22.5)	
Asian	18 (3.7)	12 (3.4)	6 (4.3)	
Other	21 (4.3)	18 (5.2)	3 (2.2)	.117
Body mass index				
<30	227 (46.7)	172 (49.4)	55 (39.9)	
30-39.99	187 (38.5)	129 (37.1)	58 (42.0)	
≥40	72 (14.8)	47 (13.5)	25 (18.1)	.136
Current/former smoker	163 (33.5)	108 (31.0)	55 (39.9)	.080
Recent travel	62 (12.8)	44 (12.6)	18 (13.0)	.881
Contact^d^	130 (26.7)	92 (26.4)	38 (27.5)	.894
Hospital^e^				
Suburban	205 (42.2)	150 (43.1)	55 (39.9)	
Urban	281 (57.8)	198 (56.9)	83 (60.1)	.581
Medical history				
Hypertension	267 (54.9)	185 (53.2)	82 (59.4)	.250
Diabetes	160 (32.9)	104 (29.9)	56 (40.6)	.031^b^
Cardiovascular disease	111 (22.8)	71 (20.4)	40 (29.0)	.056
Pulmonary disease	78 (16.0)	55 (15.8)	23 (16.7)	.923
Cancer	60 (12.3)	40 (11.5)	20 (14.5)	.451
Obstructive sleep apnea	46 (9.5)	29 (8.3)	17 (12.3)	.174
Immunosuppressed state	45 (9.3)	30 (8.6)	15 (10.9)	.488
Chronic kidney disease	42 (8.6)	30 (8.6)	12 (8.7)	>.999
Organ transplant	13 (2.7)	6 (1.7)	7 (5.1)	.057
None of the above^f^	113 (23.3)	87 (25.0)	26 (18.8)	.183
Symptom length, median (IQR), d	7.0 (4.0-9.0)	7.0 (4.0-10.0)	6.0 (4.0-7.8)	.332
Symptoms				
Cough	376 (77.4)	263 (75.6)	113 (81.9)	.168
Shortness of breath	343 (70.6)	227 (65.2)	116 (84.1)	<.001^b^
Fever	368 (75.7)	261 (75.0)	107 (77.5)	.638
Fatigue	271 (55.8)	202 (58.0)	69 (50.0)	.131
Nausea	83 (17.1)	66 (19.0)	17 (12.3)	.084
Diarrhea	158 (32.5)	120 (34.5)	38 (27.5)	.172
Vital signs in ER				
Pulse >100 beats/min	155 (31.9)	100 (28.7)	55 (39.9)	.024^b^
Respiratory rate >24/min	89 (18.3)	45 (12.9)	44 (31.9)	<.001^b^
Temperature >100.4 °F	154 (31.7)	99 (28.4)	55 (39.9)	.033^b^
Oxygen saturation <90%	104 (21.4)	42 (12.1)	62 (44.9)	<.001^b^
Chest radiograph findings				
Opacities/infiltrates/consolidation	412 (84.8)	283 (81.3)	129 (93.5)	.001^b^
ICU admission	161 (33.1)	23 (6.6)	138 (100.0)	<.001^b^
Treatment				
Oxygen supplementation	326 (67.1)	188 (54.0)	138 (100.0)	<.001^b^
Antibiotics	329 (67.7)	197 (56.6)	132 (95.7)	<.001^b^
Hydroxychloroquine	268 (55.1)	178 (51.1)	90 (65.2)	.007^b^
IL-6R inhibitor	33 (6.8)	3 (0.9)	30 (21.7)	<.001^b^
Remdesivir	9 (1.9)	0 (0.0)	9 (6.5)	<.001^b^
Days admitted, d				
<5	157 (32.3)	153 (44.0)	4 (2.9)	
5-10	160 (32.9)	150 (43.1)	10 (7.2)	
>10	169 (34.8)	45 (12.9)	124 (89.9)	<.001^b^
Clinical outcomes				
Extubated	—	—	78 (56.5)	
Discharged	399 (82.1)	347 (99.7)	52 (37.7)	<.001^b^
Died in hospital	22 (4.5)	1 (0.3)	21 (15.2)	<.001^b^

Abbreviations: ER, emergency room; ICU, intensive care unit; IL-6R, interleukin 6 receptor; IQR, interquartile range.

a Values are presented as No. (%) unless noted otherwise.

b *P* < .05.

c Race and ethnicity were collected by self-report. African American included only non-Hispanic African American.

d Self-reported contact with an individual with confirmed COVID-19.

e See Supplemental Table S1 (available online) for classification of hospitals.

f Patients who had none of the comorbidities in the 9 categories listed.

Among these hospitalized patients, 138 (28.4%) were intubated and required invasive mechanical ventilation. Intubated patients had a higher median age (65 vs 57 years, *P* < .001), male percentage (63.8% vs 52.6%, *P* = .033), and prevalence of diabetes (40.6% vs 29.9%, *P* = .031). Of the other comorbidities queried, there were no significant differences between intubated and nonintubated patients.

### Clinical Presentation

A minority of hospitalized patients endorsed recent travel (12.8%) or contact with a confirmed individual with COVID-19 infection (26.7%). The most commonly reported symptoms were cough (77.4%), fever (75.7%), shortness of breath (70.6%), and fatigue (55.8%).

As compared with nonintubated patients, intubated patients were more likely to present with shortness of breath (84.1% vs 65.2%, *P* < .001), respiratory rate >24/min (31.9% vs 12.9%, *P* < .001), temperature >100.4 °F (39.9% vs 28.4%, *P* = .033), and oxygen saturation <90% (44.9% vs 12.1%, *P* < .001). Intubated patients were also more likely to have evidence of opacities, infiltrates, or consolidation on chest radiographs (93.5% vs 81.3%, *P* = .001).

### Hospital Course

During their admission, a majority of patients infected with COVID-19 received oxygen supplementation (67.1%), antibiotics (67.7%), and hydroxychloroquine (55.1%); 161 (33.1%) were transferred to the intensive care unit (ICU); 399 (82.1%) were discharged; and 22 (4.5%) died during their admission. Among patients who were not intubated, 45 (12.9%) had a hospital stay >10 days; 347 (99.7%) were discharged; and 1 (0.3%) patient died.

**Figure 2.**
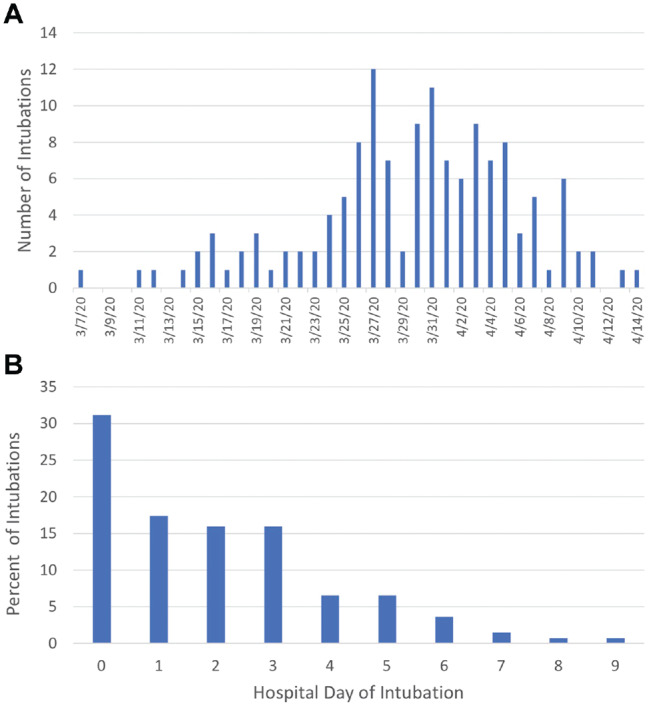
(A) Daily intubations for patients infected with COVID-19 who were admitted from March 1 to April 8, 2020. (B) Percentage of intubations by hospital day.

The first intubation for a patient infected with COVID-19 occurred on March 7, 2020 (**Figure 2A**). The number of daily intubations for patients infected with COVID-19 gradually increased, reaching a peak on March 27, 2020, with 12 intubations, and then declined in the days afterward. Forty-three intubations (31.2%) occurred within 24 hours from arrival at the hospital, and intubation rates gradually decreased on subsequent hospital days (**Figure 2B**).

A significantly higher percentage of intubated patients received oxygen supplementation, antibiotics, hydroxychloroquine, an IL-6 receptor inhibitor, or remdesivir as compared with nonintubated patients (**Table 1**). Of those intubated, 19 (13.8%) underwent tracheostomies, and 6 (4.3%) received extracorporeal membrane oxygenation (**Table 2**); 78 (56.5%) were extubated; 21 (15.2%) died; and 39 (28.3%) remained intubated at last follow-up. Six (7.7%) extubated patients and 3 (14.3%) deceased patients were intubated for >14 days. Of the 39 patients still intubated in the hospital at last follow-up, 64.1% had been intubated for >14 days.

**Table 2.** Hospital Course of Intubated Patients With COVID-19 (n = 138).

	No. (%)
Hospital days prior to intubation, d^a^	2 (0-3)
Tracheostomy	19 (13.8)
ECMO	6 (4.3)
Reintubation	16 (11.6)
Total days intubated	
Extubated (n = 78)	
1-7	30 (38.5)
7-14	42 (53.8)
>14	6 (7.7)
Deceased (n = 21)	
1-6	6 (28.6)
7-14	12 (57.1)
>14	3 (14.3)
Intubated at last follow-up^b^ (n = 39)	
1-6	2 (5.1)
7-14	12 (30.8)
>14	25 (64.1)

Abbreviation: ECMO, extracorporeal membrane oxygenation.

a Median interquartile (range).

b Last follow-up was April 18, 2020.

### Multivariable Predictive Models

**Figure 3.**
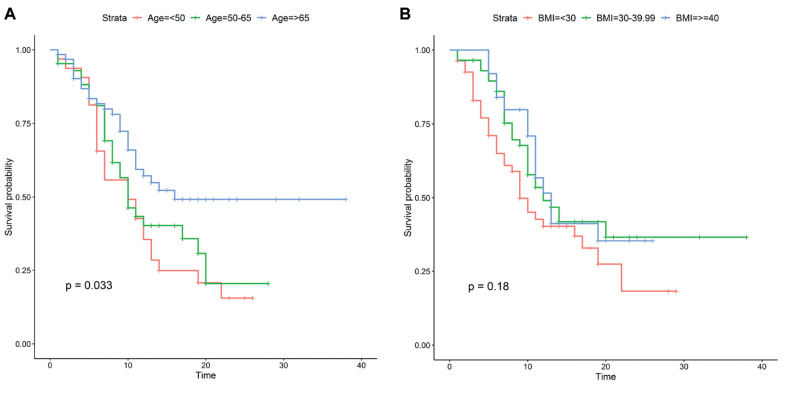
Kaplan-Meier curves for time to extubation stratified by (A) age and (B) body mass index (BMI).

**Table 3** shows the multivariable logistic regression model predicting intubation. Patients who were >60 years old (OR, 3.9; 95% CI, 2.30-6.76), were male (OR, 1.69; 95% CI, 1.04-2.77), presented with an oxygen saturation <90% (OR, 4.01; 95% CI, 2.39-6.88), had a respiratory rate >24/min (OR, 2.17; 95% CI, 1.22-3.89), had shortness of breath as a symptom (OR, 2.05; 95% CI, 1.16-3.72), or had a history of diabetes (OR, 1.64; 95% CI, 1.02-2.66) were more likely to be intubated than patients who did not have those risk factors. The Kaplan-Meier survival analysis for time to extubation, as stratified by age and BMI, suggested that only age influenced the rate of extubation (**Figure 3**). However, after adjustment for confounders in the Cox regression analysis, age >65 years was associated with a decreased chance of extubation versus age <50 years (hazard ratio [HR], 0.45; 95% CI, 0.23-0.90), and intubated patients with COVID-19 with a BMI of 30 to 39.99 (HR, 0.53; 95% CI, 0.32-0.90) or ≥40 (HR, 0.40; 95% CI, 0.19-0.82) were associated with a decreased chance of extubation versus patients with a BMI <30 (**Table 4**).

**Table 3.** Multivariable Logistic Regression Model for Intubation.

	Intubated	
Variable	Odds Ratio (95% CI)	*P* Value
Age, y		
≤ 60	1.00	
>60	3.90 (2.30-6.76)	<.001^a^
Sex		
Female	1.00	
Male	1.69 (1.04-2.77)	.034^a^
Race and ethnicity^b^		
Non-Hispanic white	1.00	
African American	0.56 (0.30-1.01)	.058
Hispanic white	0.83 (0.44-1.55)	.565
Asian/other	0.71 (0.27-1.71)	.457
Hospital^c^		
Suburban	1.00	
Urban	1.35 (0.82-2.23)	.241
Body mass index		
<30	1.00	
30-39.99	1.46 (0.87-2.46)	.151
≥40	1.92 (0.92-4.00)	.080
Respiratory rate >24/min	2.17 (1.22-3.89)	.009^a^
Temperature >100.4 °F	1.59 (0.97-2.58)	.064
Oxygen saturation <90%	4.01 (2.39-6.88)	<.001^a^
Pulse >100 bpm	1.60 (0.96-2.65)	.069
Diabetes	1.64 (1.02-2.66)	.046^a^
Shortness of breath	2.05 (1.16-3.72)	.015^a^

a *P* < .05.

b Race and ethnicity were collected by self-report. African American included only non-Hispanic African American.

c See Supplemental Table S1 (available online) for classification of hospitals.

**Table 4.** Cox Proportional Hazards Regression Model for Time to Extubation.

	Intubated	
Variable	Hazard Ratio (95% CI)	*P* Value
Age, y		
<50	1.00	
50-65	0.63 (0.34-1.18)	.145
>65	0.45 (0.23-0.90)	.024^a^
Sex		
Female	1.00	
Male	1.34 (0.82-2.20)	.236
Race and ethnicity^b^		
Non-Hispanic white	1.00	
African American	1.16 (0.58-2.34)	.675
Hispanic white	0.65 (0.36-1.19)	.161
Asian/other	1.04 (0.40-2.75)	.929
Body mass index		
<30	1.00	
30-39.99	0.53 (0.32-0.90)	.018^a^
≥40	0.40 (0.19-0.82)	.012^a^
Hypertension	0.73 (0.44-1.20)	.210
Current/former smoker	0.66 (0.39-1.13)	.130

a *P* < .05.

b Race and ethnicity were collected by self-report. African American included only non-Hispanic African American.

## Discussion

This study describes one of the largest cohorts of hospitalized patients with COVID-19 in the United States and, to the best of our knowledge, is the first to analyze for risk factors associated with intubation and time to extubation in the US population. Most large cohorts of patients with COVID-19 reported in the literature were based on Chinese and Italian populations, which previously had the most infections, although reports detailing the American experience have recently begun to emerge in Seattle, California, and New York.^4,9-12^

The rate of intubation in our study was 28.4%, which is within the range of previous studies. In China, 12% of hospitalized patients required mechanical ventilation, while in New York 12.2% to 33.1% of inpatients infected with COVID-19 were intubated.^4,11,13^ In our Chicago cohort, daily intubations for patients infected with COVID-19 gradually increased in March until peaking on March 27, 2020, which was 6 days after stay-at-home orders were announced in Illinois. The subsequent decline in daily intubations was likely related to the implementation of local mitigation policies. About a third of intubated patients were intubated upon presentation in the emergency room, a sign of the rapid clinical deterioration experienced by a subset of patients infected with COVID-19.^4^ Intubations were less frequent with each additional day that the patient was in the hospital, suggesting that the prognosis for a severe disease course may be determined within the first few days of admission. Once intubated, only 27.5% of patients were intubated for <7 days, while 90% had a hospital stay >10 days. Identifying and treating at-risk patients early in the infection may provide benefit in preventing disease progression.

On initial presentation to the emergency room, certain clinical findings were associated with the need for mechanical ventilation during hospitalization. Shortness of breath was reported by 84.1% of intubated patients and was the only symptom associated with intubation. Oxygen saturation <90% and increased respiratory rate were also predictors for intubation, which is not surprising since they are often clinical indicators for intubation. However, each of these predictors was absent in more than half of patients who were eventually intubated in our cohort. Therefore, additional risk factors likely contribute to the clinical prognosis of the patient with COVID-19.

In our study, advanced age was strongly associated with intubation and time to extubation, after adjusting for confounding factors. Age was reported as a predictor of mortality in Chinese and Italian populations. Patients at least 65 years old in Wuhan, China, were identified as being at higher risk of mortality in a prospective cohort of 179 patients.^14^ In Italy, increasing age was also associated with mortality among ICU patients.^15^ In the United States, the median or mean age of patients in the ICU or on mechanical ventilation ranges from 64 to 70 years.^4,9,10^ The pathophysiology underlying the increased risk of a more severe clinical presentation in older patients is an area of active research. COVID-19-induced reduction of ACE2 (angiotensin-converting enzyme 2), which regulates inflammation and is already present in lower levels in the elderly, has been proposed as a possible mechanism leading to higher disease severity.^16^ ACE2 is also a component of Leydig cells within the male testes, leading to a potential site of infection and viral safe harbor, which may contribute to the higher percentage of male patients with COVID-19 as compared with females.^17^ However, further investigation is necessary to elucidate how age and sex influence clinical presentation.

BMI was also a significant risk factor predictive of time to extubation among intubated patients with COVID-19. In the literature, BMI had not been initially discussed as extensively as other risk factors in the COVID-19 pandemic, likely due to obesity not being as prevalent in the Italian and Chinese populations as in the United States.^18^ The United States has one of the highest rates of obesity in the world, with 40% of adults having a BMI ≥30 and 9.2% having a BMI ≥40.^19,20^ Initial reports from New York City have suggested that obese patients may have a higher likelihood of being admitted and placed on mechanical ventilation.^4,21^ The impact of obesity on severity of disease is not surprising. Obesity is already associated with decreased pulmonary function, and with the addition of COVID-19 injuring lung tissue, adequate ventilation would inevitably become more difficult.^22^ Biologically, obesity is also associated with higher levels of inflammatory cytokines, which may be exacerbated by the release of TNF-α and IL-6 from infected pneumocytes and pulmonary cells.^23,24^

The most common comorbidities among patients in this study were hypertension, diabetes, and cardiovascular disease, which are consistent with 2 case series of hospitalized patients with COVID-19 in New York.^4,11^ Similarly, in a national Chinese study, chronic obstructive pulmonary disease, hypertension, and diabetes were identified as risk factors associated with admission to the ICU, placement on invasive mechanical ventilation, or death.^25^ Diabetes had a higher prevalence in intubated patients in our study and, after adjustment for confounding factors, was the only comorbidity found to be independently associated with intubation in our study population. While the pathophysiology of the interaction between diabetes and COVID-19 disease is unknown at this time, metabolic disorders have been associated with the impairment of macrophages and lymphocytes.^26^ As a result, diabetic patients may be at higher risk of an inadequate immune response to a COVID-19 infection. Further research is necessary to explore this possible relationship.

The overall case fatality rate in the literature ranges from 1.4% to 2.3%, while ICU mortality varies from 26% to 67%, although current reports likely underestimate overall inpatient mortality, given the unknown outcome of a high percentage of reported cases, which applies to our study as well.^1,9,10,27^ Our cohort of intubated patients had a lower case fatality rate of 15.2%, which is similar to a study from New York City, which had a case fatality rate of 14.6% among those requiring intubation and 10.2% among admitted patients.^4^ In our study, overall mortality of admitted patients was noticeably much lower than other studies at 4.5%, but that is likely attributable to our exclusion of patients with DNR/DNI status, who in general had a high mortality rate due to baseline poor clinical status and refrainment from aggressive care.

The outcomes of tracheostomy for intubated patients with COVID-19 are unclear at this time. Open tracheostomy has a high risk of transmission of COVID-19 to health workers, as it is an aerosol-generating procedure. Several guidelines and checklists have been developed to protect otolaryngologists from infection during these procedures.^28-30^ However, the optimal timing of tracheostomy in the COVID-19 population and whether there is any benefit to an early tracheostomy as seen in other diseases are unknown at this time.^31^ In our study, most deaths occurred within 2 weeks of intubation, and many patients continue to remain intubated beyond 2 weeks. A portion of these patients will very likely need tracheostomies for long-term ventilation and weaning from ventilation. The timing and outcomes of tracheostomy in the COVID-19 population require further study.

There are several limitations to this study that should be considered. First, several patients in this study were still hospitalized at the time of last follow-up; as a result, some clinical outcomes were not known, such as mortality. Second, deciding when to intubate a patient is a complex clinical decision based on many factors, and different health care providers may have different criteria for deciding when to intubate patients with COVID-19. While this study included patients treated at several hospitals, the results might not be generalizable to other institutions. Third, laboratory results were not collected and could contribute as additional predictive factors for intubation and prolonged intubation. Fourth, although data were derived directly from medical records, they were originally collected for clinical care and thus suffer from rapidly evolving practice guidelines, which may lead to bias. We utilized several statistical methods to reduce bias and confounding effects. Last, the retrospective design of this study precludes the ability to draw causal conclusions.

## Conclusion

Age, male sex, and a history of diabetes were independent risk factors associated with intubation in hospitalized patients with COVID-19 in the Chicago metropolitan area. Time to extubation was influenced by age and obesity only. Further research and data from other institutions are warranted to more accurately characterize this novel disease.

## Supplemental Material

Appendix_5.1.20 – Supplemental material for Factors Associated With Intubation and Prolonged Intubation in Hospitalized Patients With COVID-19 Click here for additional data file. Supplemental material, Appendix_5.1.20 for Factors Associated With Intubation and Prolonged Intubation in Hospitalized Patients With COVID-19 by Kevin Hur, Caroline P. E. Price, Elizabeth L. Gray, Reeti K. Gulati, Matthew Maksimoski, Samuel D. Racette, Alexander L. Schneider and Ashoke R. Khanwalkar in Otolaryngology–Head and Neck Surgery
