# Supplementary material for: Factors Associated With Intubation and Prolonged Intubation in Hospitalized Patients With COVID-19
Source: Otolaryngol Head Neck Surg. 2020 May 19;163(1):170–8. doi: 10.1177/0194599820929640 (PMC7240317; doi:10.1177/0194599820929640)
Supplement: Appendix_5.1.20 – Supplemental material for Factors Associated With Intubation and Prolonged Intubation in Hospitalized Patients With COVID-19 [file Appendix_5.1.20.docx]

**SUPPLEMENTARY APPENDIX**

**Statistical Methods**

Logistic Regression Model for Intubation

Cox Proportional-Hazards Regression Model for Time to Extubation

Predictor Variables

**Supplementary Figures**

Supplementary Figure 1. Random Forest Model

Supplementary Figure 2. Multivariable Logistic Regression Model

Supplementary Figure 3. Cross-validation of Logistic Regression Model

Supplementary Figure 4. Cox Proportional-Hazards Regression Model for Time to Extubation

**Supplementary Tables**

Supplementary Table 1. List of Northwestern Medicine Hospitals

Supplementary Table 2. Additional Characteristics of Intubated Patients with COVID-19 Infection

**Statistical Methods**

*Logistic Regression Model for Intubation*

Patients who had never been intubated and had yet to be discharged as of 4/18/2020 (n=35), and patients with missing BMI and former smoking history (n=4) were excluded from analysis. Univariate analyses for both tables (intubation and length of intubation) were performed with Wilcoxon rank sum tests for continuous variables and Chi-square or the Fisher exact test for categorical variables. Random Forest (RF) model (suppl. Figure 1) was used to predict intubation by entering 31 collected variables with 34 levels (demographic information, medical history, symptoms and vital signs on admission) into the model (1000 trees) using repeated cross-validation with 5 folds, 5 repeats, and internal down-sampling to account for imbalance between cohorts, tuning the number of variables (mtry) and using the receiver operating curve (ROC) as the metric of choice. Variable importance was computed from the RF to help select variables to include in a logistic regression (suppl. Figure 2). Logistic regressions of these variables were performed to report odds ratios, and a final subset of variables were chosen with the goal of maximizing AUC from a ROC curve created using leave-one-out cross validation (suppl. Figure 3). Continuous covariates were categorized based on clinical relevance as well as estimated univariate logit curves of the risk of intubation.

*Cox Proportional-Hazards Regression Model for Time to Extubation*

A total of 138 patients were intubated. For univariate analyses (see suppl. table 5) of intubation time < 7 days vs. 7 days or greater, patients who had not been intubated for 7 days, nor extubated or died were removed (n=2). For analyses of time from intubation to extubation, of the 138 patients who were intubated and had yet to be extubated were censored as of 4/19/2020 (n=2), and patients who died were censored on the day of their death (n=21). Univariate analyses were done using the log-rank test. Multivariate analyses were done using Cox Proportional Hazards models stratified by hospital (Northwestern downtown hospital vs. all other suburban hospitals), considering demographic information and medical history as covariates (see suppl. Figure 4). Continuous covariates were categorized based on clinical relevance and improved model performance.

Predictor variables:

Hospital (urban vs. suburban)

Age (<=60 vs. >60)

bmicat

saturation (<90 vs. >=90)

pulse (<=100 vs. >100)

rr (<=24 vs. >24)

temp (<=100.4 vs >100.4)

gender

race

travel

contact

cough

sob

fever

diarrhea

nausea

fatigue

smell

taste

rhinorrhea

congestion

dm

htn

cv

transplant

immuno

cancer

osa

pulm

ckd

formersmoke

Supplementary Figure 1. Random Forest Model

The random forest model was developed to identify variables that correlate with intubation among COVID-19 positive hospitalized patients.


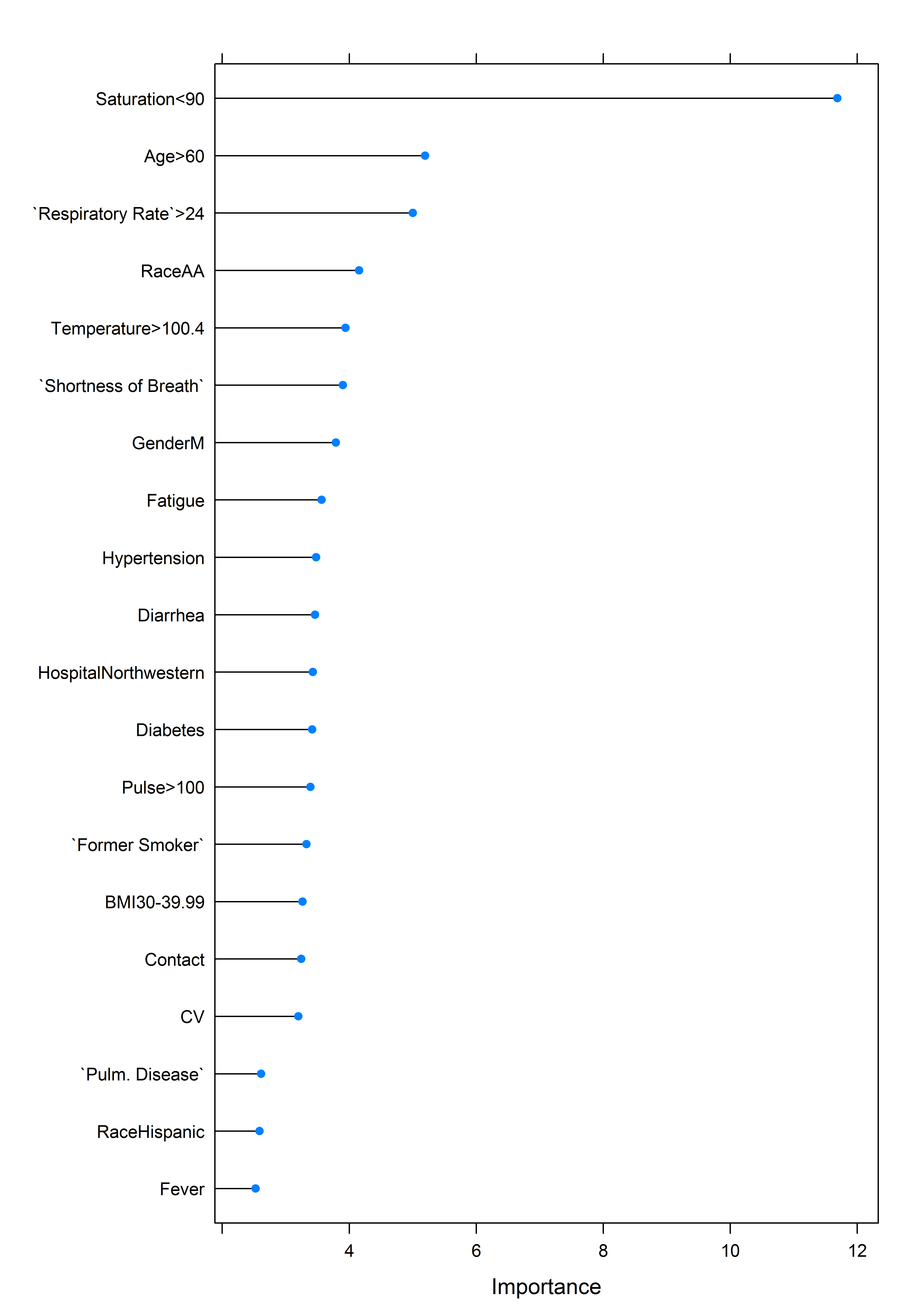


Supplementary Figure 2. Final Adjusted Logistic Regression Model

The predictor variables included in the final adjusted logistic regression model are displayed below.


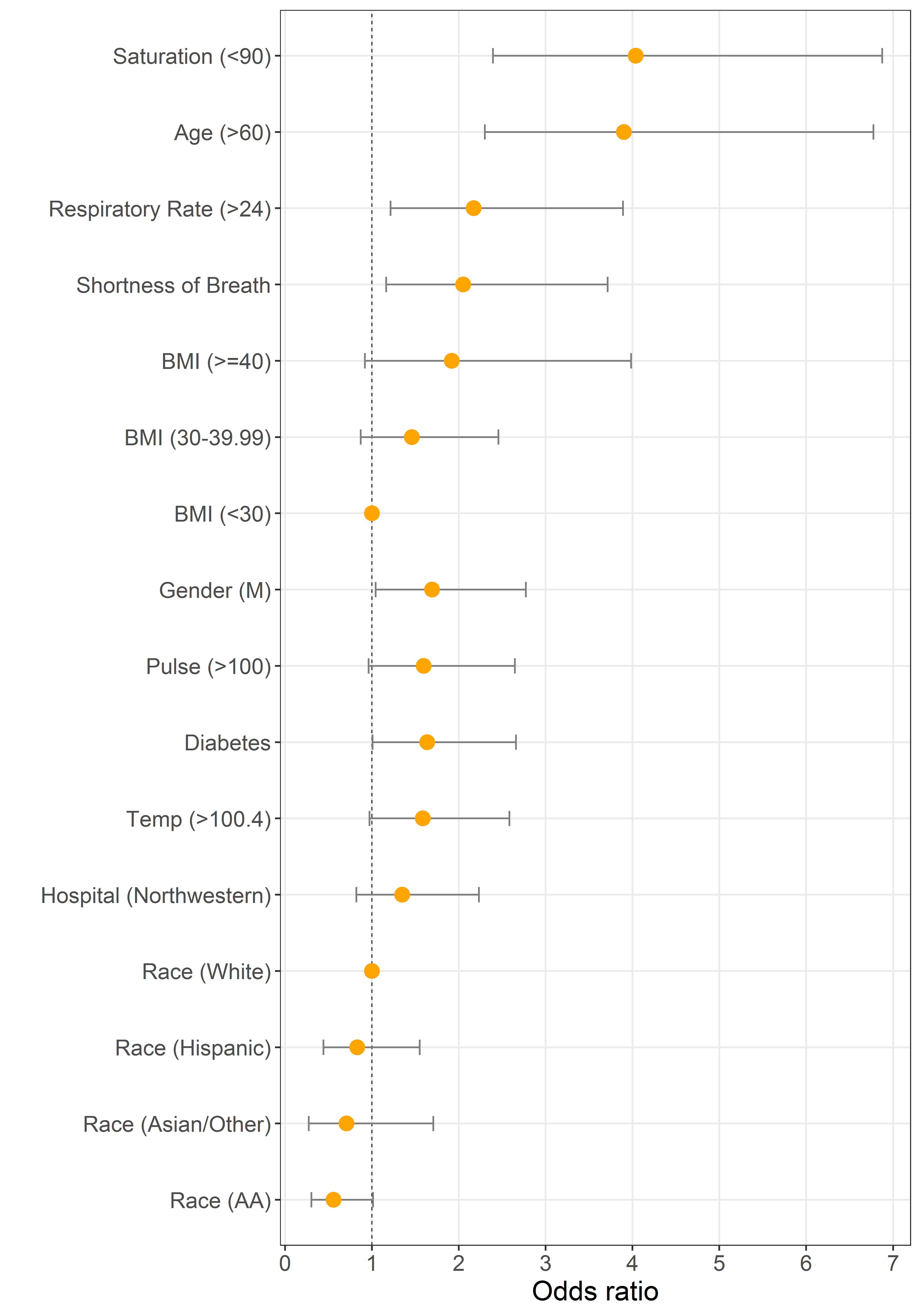


Supplementary Figure 3. Cross-Validation of the Logistic Regression Model

Discrimination of leave-one-out cross validation of the final adjusted logistic regression model was assessed using a receiver operating characteristics curve. AUC= 0.77.


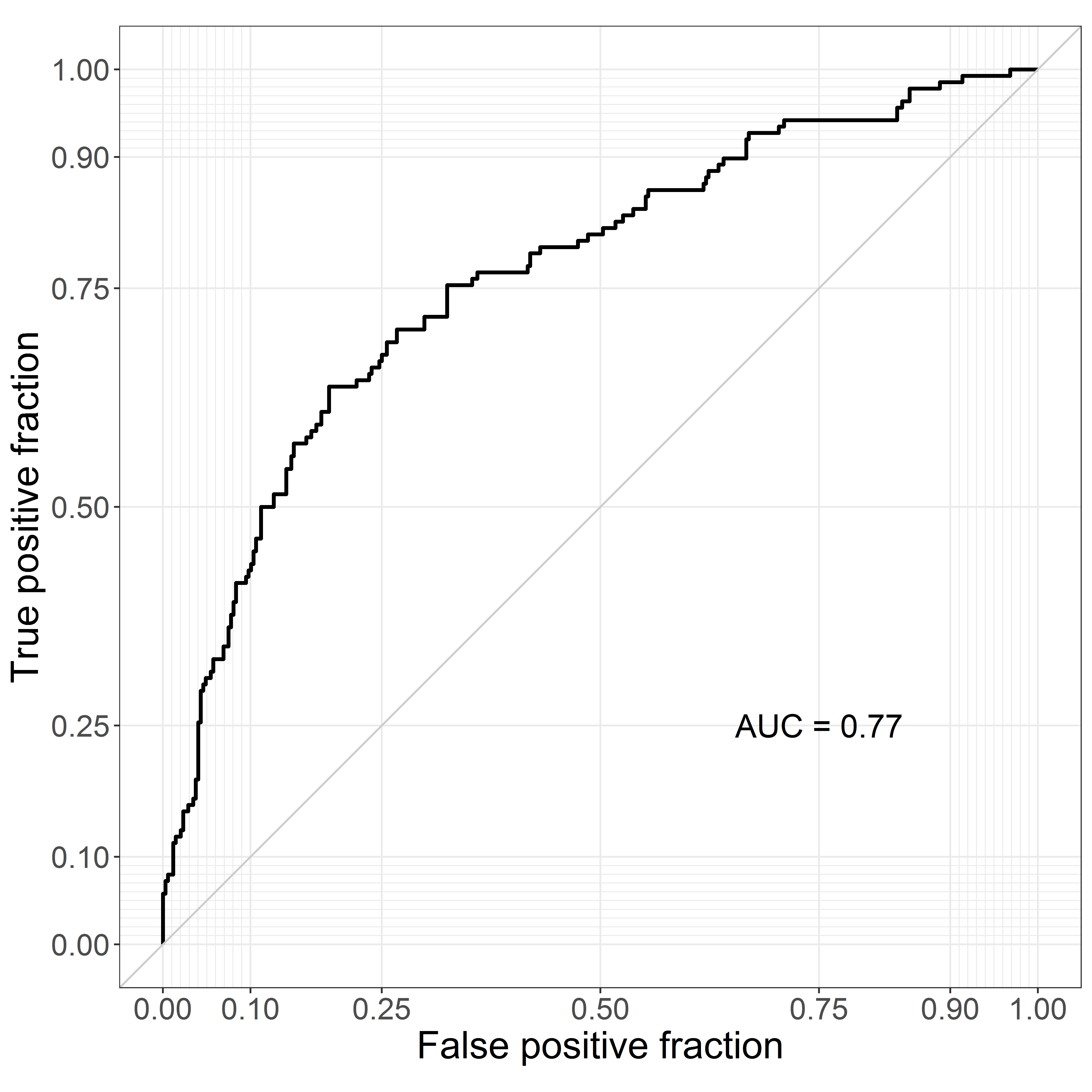


Supplementary Figure 4. Cox Proportional-Hazards Model for Time to Extubation


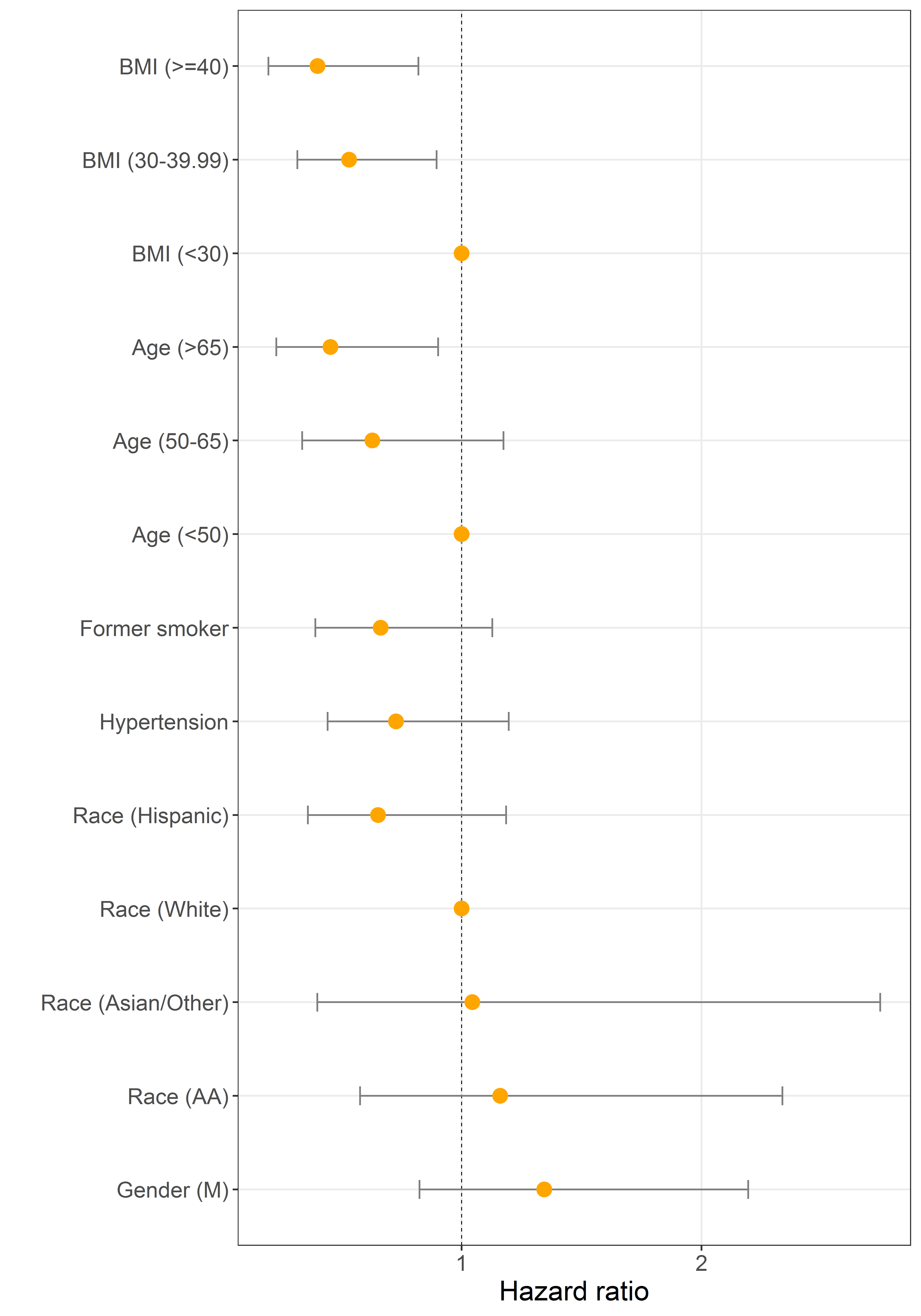


Supplementary Table 1:

Northwestern Medicine (NM) affiliated healthcare centers in the Enterprise Data Warehouse:

| **Name of Hospital** | **Beds** | **Location** | **Location Type^a^** | **No. of COVID-19 Patients^b^** |
| --- | --- | --- | --- | --- |
| NM Central DuPage Hospital | 394 | Winfield, IL | Suburban | 70 |
| NM Huntley Hospital | 128 | Huntley, IL | Suburban | 6 |
| NM Delnor Hospital | 159 | Geneva, IL | Suburban | 22 |
| NM McHenry Hospital^c^ | 179 | McHenry, IL | Suburban | 15 |
| NM Woodstock Hospital | 34 | Woodstock, IL | Suburban | 0 |
| NM Valley West Hospital | 25 | Sandwich, IL | Suburban | 2 |
| NM Kishwaukee Hospital | 98 | Dekaulb, IL | Suburban | 6 |
| NM Lake Forest Hospital | 198 | Lake Forest, IL | Suburban | 84 |
| Northwestern Memorial Hospital^c^ | 894 | Chicago, IL | Urban | 281 |
| Marianjoy Rehabilitation Hospital^d^ | 100 | Wheaton, IL | Suburban | 21 |

^a^Hospitals located in the city of Chicago were classified as urban. Hospitals located outside of the city of Chicago were classified as suburban.

^b^only counts patients included in study cohort

^c^denotes multispecialty teaching hospital

^d^includes COVID-19 patients who were previously treated at the other acute care hospitals listed

| Supplementary Table 2  Additional Characteristics of Intubated Patients with COVID-19 Infection^a^ | | | | | | |
| --- | --- | --- | --- | --- | --- | --- |
|  |  | **Intubated** | | | |  |
|  | **Total**  **N=486** | **No**  **N=348** | **Yes**  **N=138** | | ***P*** | |
| Age, median (IQR), y | 59 (47-69) | 57 (45.5-65.5) | 65 (50-72) | <0.001* | | |
| Body mass index, median (IQR) | 30.6 (26.5-35.6) | 30.0 (26.3-35.4) | 31.8 (26.6-37.5) | 0.071 | | |
| Current smoker, No. (%) | 16 (3.3) | 9 (2.6) | 7 (5.1) | 0.170 | | |
| **Symptoms, No. (%)** |  |  |  |  | | |
| Nasal congestion | 48 (9.9) | 32 (9.2) | 16 (11.6) | 0.405 | | |
| Rhinorrhea | 45 (9.3) | 35 (10.1) | 10 (7.2) | 0.389 | | |
| Hyposmia | 23 (4.7) | 21 (6.0) | 2 (1.4) | 0.033* | | |
| Dysguesia | 26 (5.3) | 22 (6.3) | 4 (2.9) | 0.179 | | |
| **Vital Signs in ER, median (IQR)** |  |  |  |  | | |
| Diastolic blood pressure, mmHg | 74 (65-84) | 75 (67-84) | 72 (62-85) | 0.140 | | |
| Systolic blood pressure, mmHg | 130 (116-143) | 130 (115.5-141) | 133.5 (117-146) | 0.077 | | |
| **Hospital** |  |  |  |  | | |
| Delnor | 22 (4.5) | 18 (5.2) | 4 (2.9) |  | | |
| Dupage | 70 (14.4) | 49 (14.1) | 21 (15.2) |  | | |
| Huntley | 6 (1.2) | 5 (1.4) | 1 (0.7) |  | | |
| Kishwaukee | 6 (1.2) | 4 (1.1) | 2 (1.4) |  | | |
| Lake Forest | 84 (17.3) | 65 (18.7) | 19 (13.8) |  | | |
| McHenry | 15 (3.1) | 7 (2.0) | 8 (5.8) |  | | |
| Northwestern Memorial | 281 (57.8) | 198 (56.9) | 83 (60.1) |  | | |
| Valley West | 2 (0.4) | 2 (0.6) | 0 (0.0) | 0.318 | | |

^a^Abbreviations: IQR (interquartile range)

*P<0.05
